# Hinged external fixation for Regan–Morrey type I and II fractures and fracture-dislocations

**DOI:** 10.1007/s10195-016-0395-x

**Published:** 2016-02-13

**Authors:** Alberto Castelli, Salvatore D’amico, Alberto Combi, Francesco Benazzo

**Affiliations:** Clinica Ortopedica e Traumatologica, IRCCS Policlinico San Matteo, Pavia, Italy; Clinica Ortopedica e Traumatologica, Università Degli Studi di Pavia, IRCCS Policlinico San Matteo, Pavia, Italy; Clinica Ortopedica e Traumatologica, IRCCS Policlinico San Matteo, via Golgi 19, 27100 Pavia, Italy

**Keywords:** Elbow fracture-dislocation, Hinged external fixator, Instability, Coronoid, Heterotopic ossification

## Abstract

Elbow fracture-dislocation is always demanding to manage due to the considerable soft-tissue swelling or damage involved, which can make an early open approach and ligamentous reconstruction impossible. The purpose of this study was to evaluate the role of elbow hinged external fixation (HEF) as a definitive treatment in patients with elbow dislocations associated with Regan–Morrey (R-M) type I and II coronoid fractures and soft-tissue damage. We treated 11 patients between 2010 and 2012 with HEF. Instability tests and standard X-ray examinations were performed before surgery and 1–3 to 3–6 months after surgery, respectively. All patients underwent a preoperative CT scan. Outcomes were assessed with a functional assessment scale (Mayo Elbow Performance Score, MEPS) that included 4 parameters: pain, ROM, stability, and function. The results were good or excellent in all 11 patients, and no patient complained of residual instability. Radiographic examination showed bone metaplasia involving the anterior and medial sides of the joint in 5 patients. HEF presented several advantages: it improves elbow stability and it avoids long and demanding surgery in particular in cases with large soft tissue damage. We therefore consider elbow HEF to be a viable option for treating R-M type I and II fracture-dislocations.

## Introduction

The isolated coronoid fracture is an unusual event and is associated in most cases with elbow dislocation. Regan and Morrey (R-M) distinguish three types of coronoid fracture, based on the involvement of the coronoid process. O’Driscoll suggested another classification [1–6], highlighted the importance of type 2 fractures, and introduced three subgroups of such fractures involving the anteriomedial facet of the coronoid, the tip, and the bone fragment where the anterior portion of the medial collateral ligament is attached. We can consider the elbow joint to be intrinsically stable in relation to the congruence between the articular bone components. The two bone columns, medial and lateral, are biomechanically important for varus-valgus stability [7]. The forces that induce posterior dislocation of the ulna on the humerus following an axial load are opposed by the coronoid [8]. Most elbow dislocations result in medial collateral ligament (MCL) and lateral collateral ligament (LCL) complex injury. MCL is the primary stabilizer of the elbow in valgus stress and the radial head is the secondary stabilizer. On the coronoid, we have the insertion of the anterior bundle of the ulnar collateral ligament, the anterior capsule, and the insertion of the brachialis muscle. The insertion of the MCL is on average 5 mm distal and medial to the coronoid edge [9]. There are two pathogenic mechanisms for posterior dislocation: posterolateral rotatory valgus stress [4], in which the first lesion concerns the LCL; and posteromedial varus stress, in which coronoid fracture of the anteromedial facet is characteristic [5, 7] and the elbow is less stable after closed reduction [1, 6, 7, 10]. Our goal is to validate a new approach to the treatment of elbow dislocation with coronoid fracture (R-M types 1–2 and O’Driscoll type 2) that involves applying the HEF to treat the coronoid fracture and ligament lesions.

## Materials and methods

Between 2010 and 2012, we treated 11 patients with complex elbow dislocations: 8 men and 3 women with a mean age of 41 years. The mean time to surgery was 3, 4 days (1–15) (Table 1).

**Table 1:** Summary of injury classification, results and complications

Patient	Classification	ROM at 5 weeks	Complication	Time to surgery (gg)	Bone metaplasia
A.M. 30 M	Regan 1	10–110		15	Yes
G.P.39 F	Regan 1	0–130	Ulnar n. paresthesia	1	
K.A.52 F	Regan 2	20–120		3	Yes
A.P 31 M	Regan 2	20–130		2	Yes
G.B. 45 M	Regan 2	0–130	Untightening clamp	3	
A.A.41 M	Regan 2	0–130		1	
P.P. 47 M	Regan 2	0–130		2	
F.A. 28 F	Regan 2	0–130	Mild initial pain	2	
B.R. 34 M	Regan 2	0–130		3	
G.M. 56 M	Regan 2	10–110		4	Yes
A.R. 51 M	Regan 2	20–130		2	Yes

Inclusion criteria were elbow dislocation with isolated coronoid R-M type II fracture or type I fracture with significant instability (following the O’Driscoll algorithm [10]). Exclusion criteria were R-M type III fracture, radial head fracture, and humeral condyle fracture. All patients underwent clinical examination after closed reduction (ROM, lateral pivot shift test, varus-valgus stress), preoperative X-ray examination, and CT scan; they then underwent clinical and radiographic follow-up evaluations at 1, 3, and 6 months.

## Results

Patients were evaluated at last follow-up with MEPS. The average score was 94 (9 patients had excellent and 2 had good results). The ROM achieved at the removal of the HEF (after an average of 5 weeks) was better than the elbow functional ROM (30–130°) in 9 cases. The average extension deficit was 7° (0–20°) and the average flexion was 125° (110–130°). We did not find residual elbow instability. The pain was mild in 8 patients during the first 2 weeks of mobilization, but no patient complained of pain after 6 months. We had no cases of coronoid nonunion and 2 cases of osteoarthritic joint degeneration that were not related to the good functional outcomes. There were 5 cases of bone metaplasia formation within the anterior capsule and collateral ligament complex. We did not encounter any major complications.

## Discussion

**Fig. 1:**
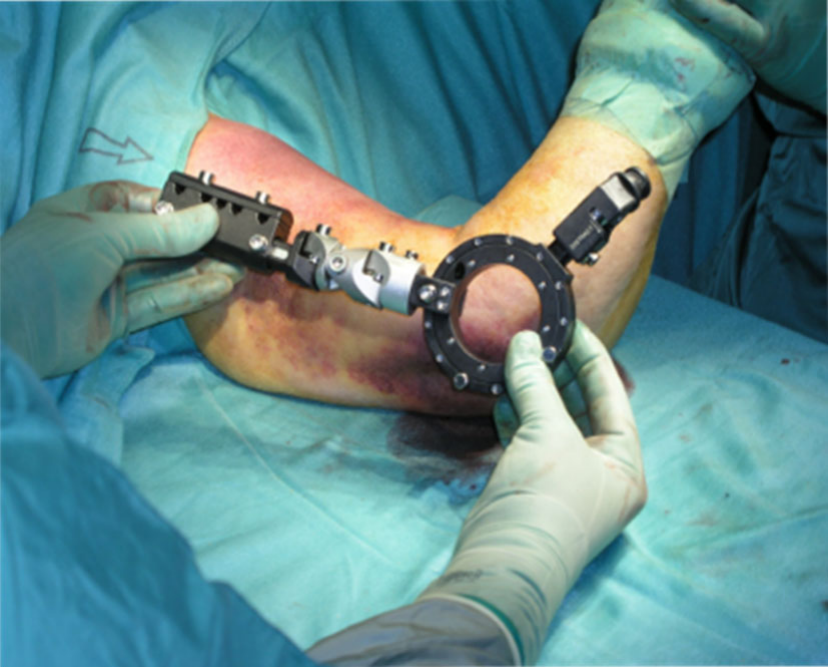
HEF placement

**Fig. 2:**
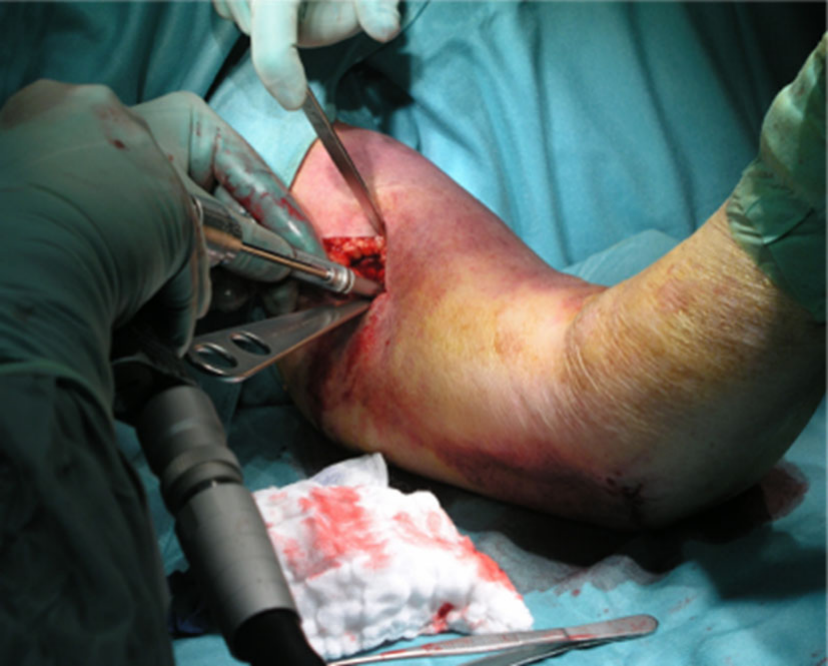
Humeral bone screws placement

**Fig. 3:**
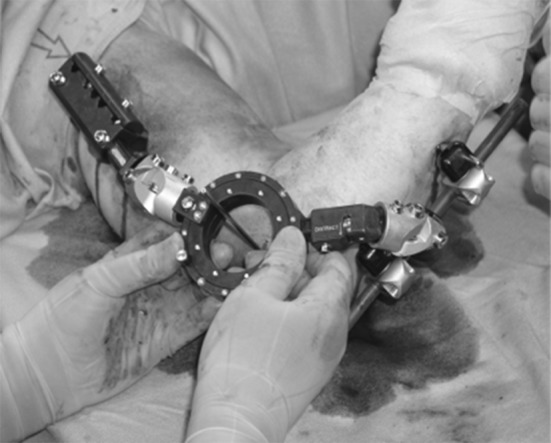
Elbow's center of rotation identification

**Fig. 4:**
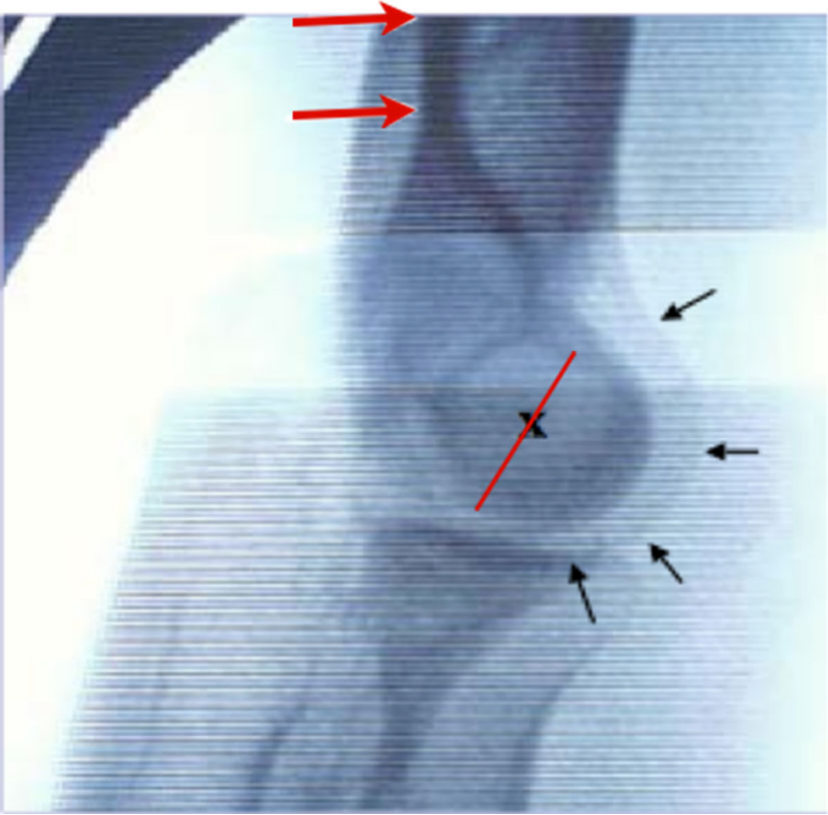
Image intensifier identification of center of rotation landmarks

**Fig. 5:**
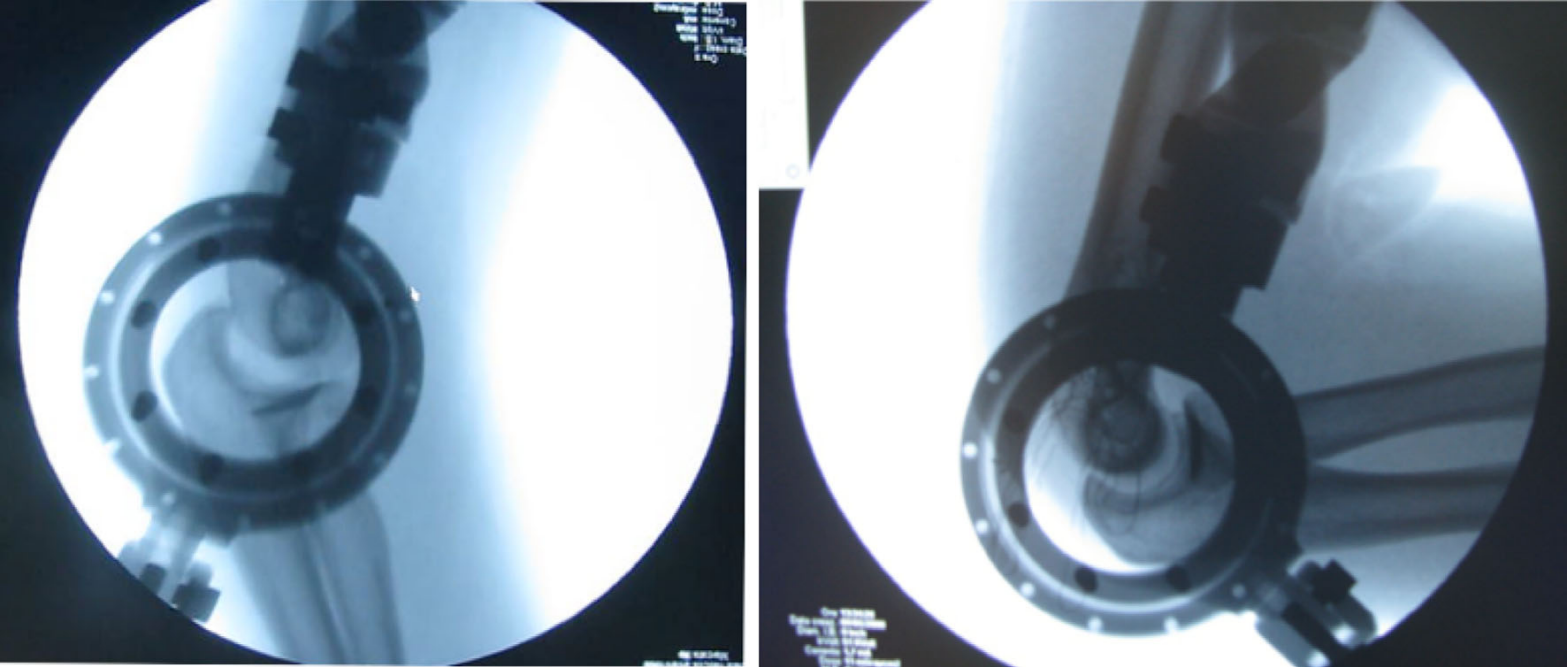
Image intensifier aids HEF placement

**Fig. 6:**
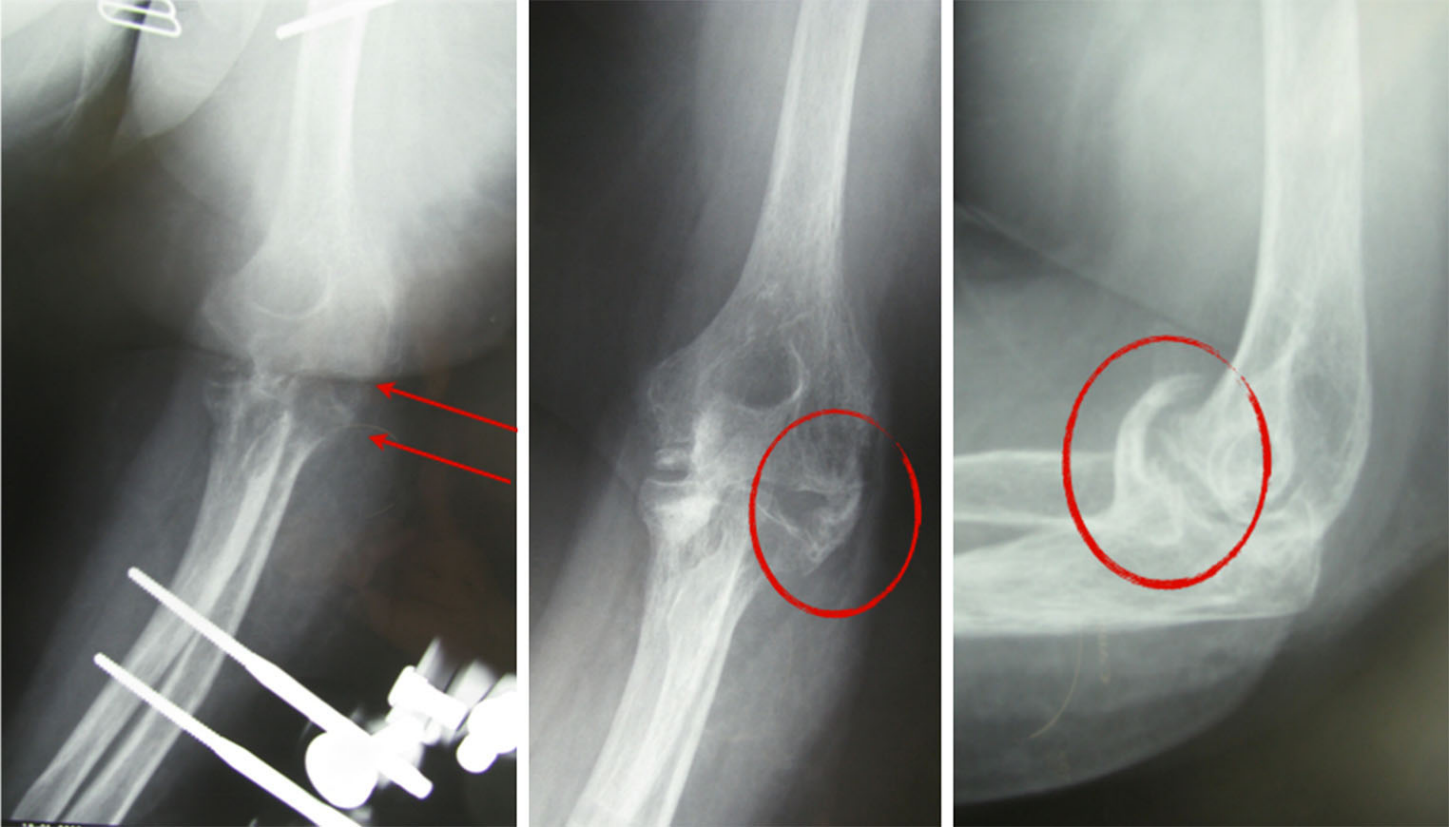
X-ray demonstrate bone metaplasia formation within the anterior capsule and collateral ligament complex

The application of elbow EF reportedly yields encouraging results [12], but it was also associated with a high rate of complications (40–50 %): screw breakage, infection, residual instability, and nerve damage [10, 11]. There are no studies in which HEF was used alone to treat complex elbow dislocation without other surgical procedures. It has usually been applied to support ORIF or ligamentous repair [12]. A misplaced HEF results in increased strength and friction during elbow mobilization, increased bending stress in the bone screws, and asymmetric tension in collateral ligaments during joint movement (Figs. 1 , 2, 3), which may be responsible for the complications [6, 10–13]. The elbow joint does not have a hinged single axis [14]. The instantaneous center of rotation of the elbow has a maximum diameter of about 3 mm, hence the importance of determining the center of rotation. Precise bone landmarks are required to identify the axis of the elbow. In the sagittal plane, concentric radiopaque circles that focus on the axis corresponding to the projection of the capitulum humeri and the medial margin of the trochlea [15, 16] as well as an opaque line along the distal humeral metaphysis are the most important landmarks (Figs. 4, 5). This landmark is due to the overlap of the medial and lateral humeral cortex, and it projects an approximate 73:27 anterior:posterior humeral cortex ratio. Several authors have argued that MCL reconstruction is rarely necessary after complex dislocations of the elbow [7, 9, 17–19]. Moreover, MCL reconstruction involves a medial dissection and ulnar nerve mobilization. We argue that indirect stabilization of the coronoid fracture by HEF allows it to heal and consolidate. During elbow valgus stress with a damaged MCL, the radial head becomes the primary stabilizer, and our cases do not include associated radial head fractures. Surgical repair of MCL, according to the literature, is considered only for injuries to athletes. The LCL complex of the elbow plays an important role as a lateral stabilizer in both flexion and extension; because of this, many authors consider ulnar collateral ligament (LCUL) repair to be essential after fracture-dislocation of the elbow [5]. Saunders claims that injury to it causes posterolateral instability. Dunning argues that only injuries to both the LCUL and the RCL (radial collateral ligament) lead to posterolateral instability [19–23]. We believe in achieving good lateral ligament complex healing with HEF protection. Even Ivo et al. used HEF without collateral ligament reconstruction for complex elbow dislocations [24]. HEF also stabilizes the elbow against varus stress during shoulder abduction due to the weight of the forearm during rehabilitation [15, 25]. We noted the formation of calcifications arranged mostly along the anterior capsule and collateral ligament complexes in follow-up X-ray examinations (Fig. 6). We do not consider them to be heterotopic ossifications that cause functional limitation. We believe that this bone metaplasia is an expression of the intraligamentous ossification that occurs during the ligament-healing process, resulting in the formation of scar tissue that is strong but less elastic than the normal ligament. This healing process happens when elbow motion and ligament isometry is provided by the EF. In order to guarantee the isometry of the collateral ligaments, it is very important to identify the center of rotation of the elbow. This treatment approach is based on simple principles:

- EF provides stability to the elbow joint, avoiding the need for open surgical approaches that can cause retracting fibrosis and heterotopic calcifications
- Early elbow mobilization limits scar retraction and supports intraligamentous bone metaplasia, while correctly centered HEF provides MCL and LCL isometry.

We believe that HEF alone could be a viable option for treating elbow dislocations associated with R-M type 1–2 fractures. However, further experience and extended case studies are required to compare the outcomes of HEF, static EF, and fixed bracing.
